# Interaction of Vitamin D Supplements and Marine *n*-3 Fatty Acids on Digestive Tract Cancer Prognosis

**DOI:** 10.3390/nu16070921

**Published:** 2024-03-22

**Authors:** Soichiro Fukuzato, Hironori Ohdaira, Yutaka Suzuki, Mitsuyoshi Urashima

**Affiliations:** 1 Division of Molecular Epidemiology, Jikei University School of Medicine, Tokyo 105-8461, Japan; sunshine1.324@jikei.ac.jp; 2 Department of Surgery, International University of Health and Welfare Hospital, Narita-shi 286-0048, Japan; ohdaira@iuhw.ac.jp (H.O.); yutaka@iuhw.ac.jp (Y.S.)

**Keywords:** vitamin D, *n*-3 PUFA, DHA, EPA, digestive tract cancers

## Abstract

A meta-analysis suggested that marine *n*-3 polyunsaturated fatty acids (PUFAs), e.g., eicosapentaenoic acid (EPA) and docosahexaenoic acid (DHA), might reduce cancer mortality. However, a randomized clinical trial of marine *n*-3 PUFA and vitamin D supplementation failed to verify this benefit. This study aimed to investigate the potential interaction between vitamin D supplementation and serum EPA and DHA levels. This post hoc analysis of the AMATERASU trial (UMIN000001977), a randomized controlled trial (RCT), included 302 patients with digestive tract cancers divided into two subgroups stratified by median serum levels of EPA + DHA into higher and lower halves. The 5-year relapse-free survival (RFS) rate was significantly higher in the higher half (80.9%) than the lower half (67.8%; hazard ratio (HR), 2.15; 95% CI, 1.29–3.59). In the patients in the lower EPA + DHA group, the 5-year RFS was significantly higher in the vitamin D (74.9%) than the placebo group (49.9%; HR, 0.43; 95% CI, 0.24–0.78). Conversely, vitamin D had no effect in the higher half, suggesting that vitamin D supplementation only had a significant interactive effect on RFS in the lower half (*p* for interaction = 0.03). These results suggest that vitamin D supplementation may reduce the risk of relapse or death by interacting with marine *n*-3 PUFAs.

## 1. Introduction

Inuit populations residing in Greenland exhibit a lower incidence of coronary artery disease, a phenomenon attributed to their consumption of fish and whales rich in marine *n*-3 PUFAs, specifically EPA and DHA [1]. In fact, the large-scale VITAL RCT, with 25,871 participants randomly assigned to either a marine *n*-3 PUFA group or placebo group, confirmed a risk reduction for myocardial infarction with *n*-3 PUFAs [2]. High blood levels of marine *n*-3 PUFAs, i.e., EPA and DHA, were also associated with reduced cancer mortality in a meta-analysis of 17 prospective cohort studies [3]. However, in the VITAL RCT, marine *n*-3 PUFA supplementation did not reduce cancer mortality [2]. These lines of evidence suggest that while serum levels of marine *n*-3 PUFAs are associated with prognoses in cancer patients, this association does not necessarily mean a causal relationship.

In contrast, there is more substantial evidence regarding the relationship between vitamin D supplementation and cancer mortality. In the AMATERASU trial, a RCT of vitamin D supplementation in patients with digestive tract cancers, the 5-year RFS rate was 77% with vitamin D supplementation vs. 69% with placebo [4]. Additionally, in the SUNSHINE trial in patients with metastatic colorectal cancer, the median progression-free survival (PFS) was 13.0 months with high-dose vitamin D3 vs. 11.0 months with standard vitamin D dosing [5]. Similarly, in the VITAL trial, cancer-related mortality occurred in 112 participants in the vitamin D group and 149 in the placebo group, demonstrating that vitamin D supplementation might potentially decrease cancer mortality by up to 25% [6]. Furthermore, in a secondary analysis of the VITAL trial, vitamin D supplementation was associated with a reduced incidence of advanced (metastatic or fatal) cancer, with the most significant risk reduction observed in normal weight individuals [7]. Several meta-analyses have also shown that vitamin D supplementation can reduce cancer mortality [8,9,10] and all-cause mortality [11]. However, as of now, no RCT has definitively proved the ability of vitamin D supplementation to suppress cancer relapse or death through a pre-planned overall analysis.

A previous study focusing on autoimmune diseases as outcomes in the VITAL trial discovered that simultaneous use of marine *n*-3 PUFAs and vitamin D supplements was able to decrease the occurrence of rheumatoid arthritis [12]. However, there is limited evidence to demonstrate an interaction between vitamin D supplements and serum levels of marine *n*-3 PUFAs. Therefore, we conducted this post hoc analysis to explore potential interactions between vitamin D supplementation and serum marine *n*-3 PUFAs (EPA + DHA) levels, utilizing residual samples from the AMATERASU trial. 

Moreover, since several studies have suggested that *n*-6 PUFAs are a risk factor for the development of breast cancer and colorectal cancer [13,14,15,16], we also investigated *n*-6 PUFAs in addition to *n*-3 PUFAs.

## 2. Materials and Methods

### 2.1. Trial Design

This analysis was conducted retrospectively on data from the AMATERASU trial (UMIN000001977), a randomized, double-blind, placebo-controlled study carried out at the International University of Health and Welfare Hospital in Otawara, Tochigi, Japan, between January 2010 and February 2018. The trial enrolled 417 patients aged 30 to 90 with stage I to III digestive tract cancer. Participants were randomly assigned, in a ratio of 3:2, to receive either vitamin D3 supplementation (2000 IU/day) or a placebo, starting approximately two weeks after surgery. Approval for the trial protocol was obtained from the ethics committees of both the International University of Health and Welfare Hospital (ethics approval code: FK-1; approved on 4 December 2009) and the Jikei University School of Medicine in Nishi-shimbashi, Tokyo, Japan (ethics approval code: 21–216 [6094]; approved on 1 January 2010). Before surgery, all participants provided written consent individually.

### 2.2. Participants

Out of the 417 participants enrolled in the AMATERASU trial and randomly allocated to either the vitamin D supplement group (n = 251) or the placebo group (n = 166), PUFA levels were assessed in residual serum samples from 302 patients who were followed up for a median duration of 3.3 years (interquartile range: 2.3–5.2 years; maximum: 7.6 years). The original report contains further information regarding the inclusion and exclusion criteria.

### 2.3. Outcome

The primary outcome was relapse or death. Relapse time was defined as the time from starting supplementation to the date when outcomes occurred or the patient was censored.

### 2.4. Serum PUFA Measurement by Gas Chromatography

Blood samples were collected about two weeks after surgery (before initiating vitamin D supplementation) and about 1 year after initiating vitamin D supplementation. Quantitative measurements of three types of PUFAs, namely EPA, DHA, and arachidonic acid (AA), were conducted by SRL, Inc. (Shinjuku, Tokyo, Japan) using reagent TC-70 and gas chromatography. The combined value of EPA plus DHA levels was considered as marine *n*-3 PUFAs, while AA served as the control for *n*-3 PUFAs because it is an *n*-6 PUFA.

### 2.5. Statistical Analysis

The subjects were divided into higher and lower half groups for EPA, DHA, EPA + DHA, and AA based on the cutoff at their respective median values. Patient characteristics were compared between vitamin D and placebo groups, as well as between higher and lower half EPA + DHA groups. Continuous variables were evaluated by Mann–Whitney tests and categorical variables by the chi-square test. For the survival analyses, 5-year RFS was compared between the higher and lower EPA + DHA value groups, as well as between the vitamin D and placebo groups. Additionally, Nelson–Aalen cumulative hazard curves were generated to illustrate changes in the risk of relapse or death over time. HRs and corresponding 95% confidence intervals (CIs) were calculated using a Cox proportional hazards model. To investigate the interaction between the vitamin D supplementation and the level of serum fatty acids, the *p* for this interaction was analyzed including three variables, for example, vitamin D group, the subgroup of patients with low levels of serum EPA + DHA, and both multiplied together. A *p* value of <0.05 was considered statistically significant. All data were analyzed using Stata 18.0 software (StataCorp LP, College Station, TX, USA).

## 3. Results

### 3.1. Study Population

**Figure 1:**
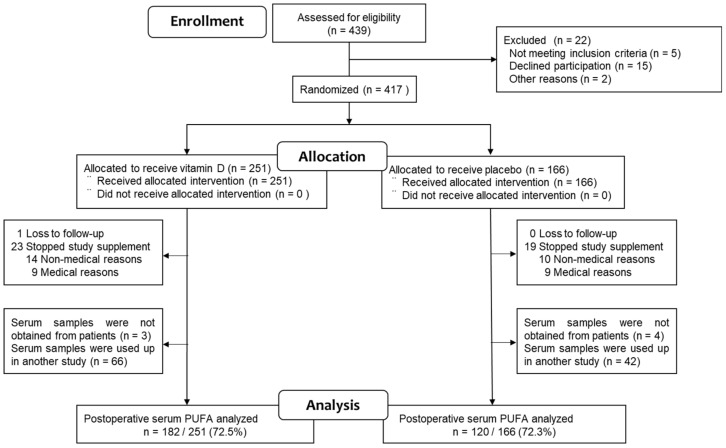
Study flowchart.

A total of 417 patients with digestive tract cancers were initially enrolled in the study, with 251 (60%) assigned to receive vitamin D supplementation and 166 (40%) assigned to the placebo group. Subsequently, one patient in the vitamin D group was lost to follow-up, and serum samples could not be collected from seven patients (three from the vitamin D group, four from the placebo group). Additionally, 113 serum samples were depleted due to use in another study (66 from the vitamin D group and 42 from the placebo group). Consequently, serum samples suitable for PUFA measurement were available for 302 (72.4%) of the original participants in the AMATERASU trial (182 from the vitamin D group and 120 from the placebo group) (Figure 1).

The study population consisted of 204 males (67.6%) and the mean age was 66 years (range, 35–90). The patients’ cancer types and stages were as follows: esophageal cancer, 7.6%; gastric cancer, 45.4%; small bowel cancer, 0.3%; colorectal cancer, 46.7%; stage I, 91.4%; stage II, 7.3%; and stage III, 1.3%. The median follow-up time was 3.3 years (interquartile range [IQR], 2.3–5.2 years). 

### 3.2. Comparison of Patient Characteristics

Table 1 shows a comparison of the patient characteristics between the groups receiving vitamin D and the placebo. Notably, there were no disparities in patient characteristics, including baseline PUFA levels and those after 1 year, between the two groups.

A comparison of the characteristics of patients stratified based on the median serum levels of EPA + DHA into higher and lower halves are shown in Table 2. The median (IQR) baseline 25(OH)D level in the higher half was 24 ng/mL (18–30), which was significantly greater than the value of 19 ng/mL (14–24) observed in the lower half (*p* < 0.0001). Likewise, the 25(OH)D insufficiency (<20 ng/mL) rate at baseline was 29.3% in the higher half, which was significantly less than the rate of 55.6% in the lower half (*p* < 0.001).

At 1 year after treatment in the placebo group, the rate of 25(OH)D insufficiency remained significantly lower in the higher half at 34.0%, compared to 61.5% in the lower half (*p* = 0.005). However, at 1 year after supplementation in the vitamin D group, the rate of 25(OH)D insufficiency was only 7.3% in the higher half, which was equivalent to the rate of 6.7% observed in the lower half.

Additionally, the AA levels were also higher in the higher half. Although the patients in the higher half were older compared to the lower half, the other characteristics remained consistent between them.

### 3.3. Risk of Relapse or Death in Lower Compared to Higher Half of PUFA

**Figure 2:**
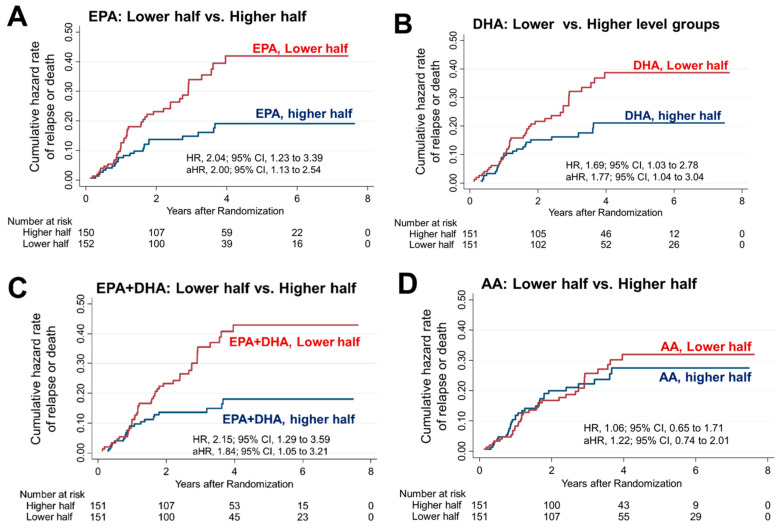
Risk of relapse or death in the higher vs. lower PUFA group. Nelson–Aalen cumulative hazard curves comparing relapse or death in the higher and lower halves for (**A**) EPA, (**B**) DHA, (**C**) EPA + DHA, and (**D**) AA levels. HRs were adjusted for vitamin D supplementation, age, sex, serum 25(OH)D levels, cancer site, stage and adjuvant chemotherapy, and are presented as adjusted HRs (aHR) with 95% CIs. The group with lower half of EPA, DHA or EPA + DHA showed statistically significant higher risks of relapse or mortality.

In the higher EPA group (n = 150), only 23 patients (15.3%) experienced relapse or death, which was significantly lower than the 44 patients (29.0%) in the lower EPA group (n = 152). The 5-year RFS rate was significantly worse in the lower EPA group (65.6%) than the higher EPA group (82.5%; HR, 2.04; 95% CI, 1.23–3.39) (Figure 2A). This worse survival in the lower EPA group remained significant even after adjusting for vitamin D supplementation, age, sex, serum 25(OH)D levels, cancer site, stage, and adjuvant chemotherapy (HR, 2.00; 95% CI, 1.13–3.54). Similarly, the relapse or death rate was significantly lower in the higher DHA group (25 patients, 16.6%) than the lower DHA group (42, 27.8%). The 5-year RFS rate was higher in the higher (80.9%) than the lower DHA group (67.8%; HR, 1.69; 95% CI, 1.03–2.78) (Figure 2B). This difference remained significant even after adjusting for the same variables (HR, 1.77; 95% CI, 1.04–3.04). Next, the relapse and death rate in the higher EPA + DHA group (22 patients, 14.6%) was significantly lower than that of the lower EPA + DHA group (45 patients, 29.8%). The 5-year RFS was higher in the higher (80.9%) than the lower EPA + DHA group (67.8%; HR, 2.15; 95% CI, 1.29–3.59) (Figure 2C). The difference remained significant even after adjusting for the same variables (HR, 1.84; 95% CI, 1.05–3.21).

In contrast, in the higher AA group, relapse or death occurred in 32 patients (21.2%), which was not significantly different from that in the lower AA group (35 [23.2%]). The 5-year RFS rate of 75.8% in the higher AA group was also not different from that of the lower AA group (72.5%; HR, 1.06; 95% CI, 0.65–1.71) (Figure 2D).

### 3.4. Interaction between Lower Half of PUFA Levels and Vitamin D Supplementation

**Figure 3:**
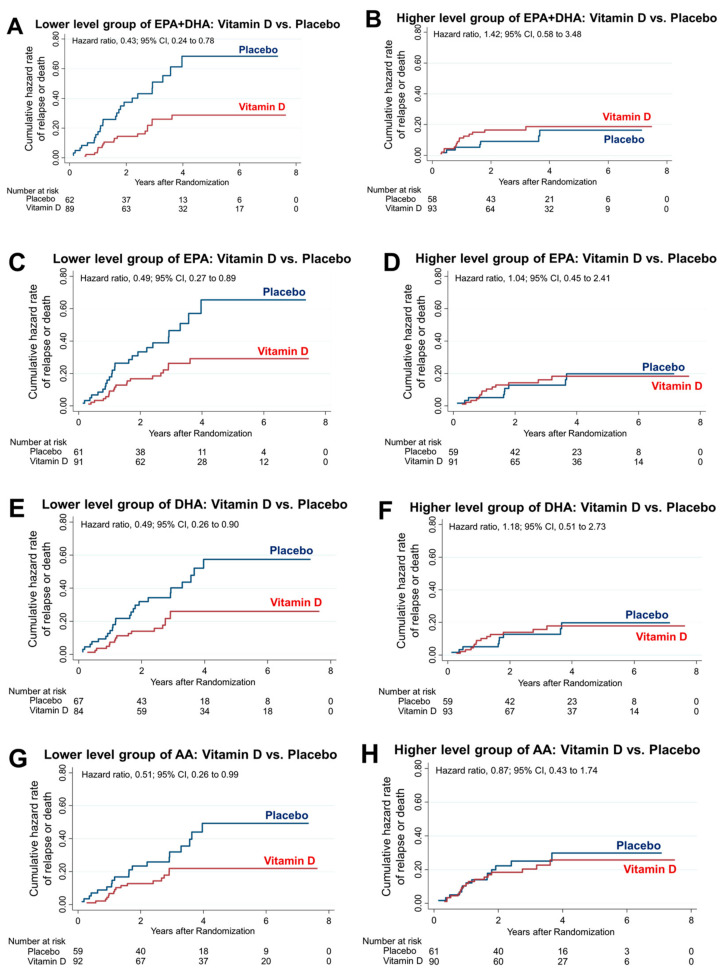
PUFAs and vitamin D interaction. Nelson–Aalen hazard curves comparing relapse or death in patients with vitamin D supplementation versus those with placebo administration in the lower and higher EPA + DHA (**A**,**B**), EPA (**C**,**D**), DHA (**E**,**F**) and AA (**G**,**H**) groups. Interaction comparisons were made between the lower and higher EPA + DHA, EPA, DHA, and AA groups. There was only a significant interaction between vitamin D supplementation and the lower EPA + DHA group (*p* for interaction = 0.03).

Among the 151 patients in the lower EPA + DHA group, relapse or death occurred in 21.4% of the patients in the vitamin D group and 41.9% of the patients in the placebo group; the 5-year RFS was significantly higher in the vitamin D group than the placebo group (24 patients [74.9%] in the vitamin D group vs. 8 patients [50.0%] in the placebo group; HR, 0.43; 95% CI, 0.24–0.78) (Figure 3A). Conversely, among the 151 patients in the higher EPA + DHA group, relapse or death occurred in 16.1% of the patients in the vitamin D group and 12.1% of the patients in the placebo group; there was no significantly difference in the 5-year RFS (24 patients [82.9%] in the vitamin D group vs. 15 patients [84.7%] in the placebo group; HR, 1.42; 95% CI, 0.58–3.48) (Figure 3B). There was a significant interaction between vitamin D supplementation and the lower EPA + DHA group (*p* for interaction = 0.03). Among the 152 patients in the lower EPA group, relapse or death occurred in 22.0% of the patients in the vitamin D group and 39.3% of the patients in the placebo group; the 5-year RFS was significantly higher in the vitamin D group than the placebo group (22 patients [74.6%] in the vitamin D group vs. 6 patients [51.4%] in the placebo group; HR, 0.49; 95% CI, 0.27–0.89) (Figure 3C). Conversely, among the 150 patients in the higher EPA group, relapse or death occurred in 15.4% of the patients in the vitamin D group and 15.3% of the patients in the placebo group; there was no significantly difference in the 5-year RFS (26 patients [83.2%] in the vitamin D group vs. 1 patient [81.8%] in the placebo group; HR, 1.04; 95% CI, 0.45–2.41) (Figure 3D). There was no significant interaction between vitamin D supplementation and the lower EPA group (*p* for interaction = 0.15). Among the 151 patients in the lower DHA group, relapse or death occurred in 20.2% of the patients in the vitamin D group and 37.3% of the patients in the placebo group; the 5-year RFS was significantly higher in the vitamin D group than the placebo group (26 patients [76.9%] in the vitamin D group vs. 12 patients [55.8%] in the placebo group; HR, 0.49; 95% CI, 0.26–0.90) (Figure 3E). Conversely, among the 151 patients in the higher DHA group, relapse or death occurred in 17.4% of the patients in the vitamin D group and 15.1% of the patients in the placebo group; there was no significantly difference in the 5-year RFS (27 patients [83.6%] in the vitamin D group vs. 17 patients [81.8%] in the placebo group; HR, 1.18; 95% CI, 0.51–2.73) (Figure 3F). There was no significant interaction between vitamin D supplementation and the lower EPA group (*p* for interaction = 0.96). Among the 151 patients in the lower AA group, relapse or death occurred in 17.4% of the patients in the vitamin D group and 32.2% of the patients in the placebo group; the 5-year RFS was significantly higher in the vitamin D group than the placebo group (28 patients [80.2%] in the vitamin D group vs. 12 patients [60.7%] in the placebo group; HR, 0.51; 95% CI, 0.26–0.99) (Figure 3G). Conversely, among the 151 patients in the higher AA group, relapse or death occurred in 20.0% of the patients in the vitamin D group and 23.0% of the patients in the placebo group; there was no significantly difference in the 5-year RFS (20 patients [77.2%] in the vitamin D group vs. 11 patients [74.0%] in the placebo group; HR, 0.87; 95% CI, 0.43–1.74) (Figure 3H). However, the interaction between vitamin D supplementation and the subgroup of patients with lower AA levels was not significant (*p* for interaction = 0.28). 

## 4. Discussion

This post hoc analysis of the AMATERASU trial revealed that vitamin D supplementation reduced the risk of relapse or death exclusively in the lower EPA + DHA group, with no significant effects in the higher group. Furthermore, this analysis identified a significant interaction between vitamin D supplementation and marine *n*-3 PUFAs in terms of prognosis in digestive tract cancer patients. To our knowledge, no previous study has indicated that vitamin D supplementation is effective for lowering the risk of cancer relapse or death in patients with lower marine *n*-3 PUFA levels.

What is the possible mechanism for this? As shown in Table 1, vitamin D supplementation did not affect the levels of PUFA one year later. However, Table 2 reveals a significant association between the lower EPA + DHA group and the prevalence of vitamin D insufficiency (25(OH)D <20 ng/mL). Furthermore, among those in the lower EPA + DHA group, in the placebo group, the number of vitamin D deficiencies at baseline and after one year were similar, while in the vitamin D supplementation group, the number of vitamin D deficiencies after one year appeared to improve compared to baseline. This pattern suggests that supplementation with vitamin D reduces the prevalence of vitamin D insufficiency more effectively in the lower EPA + DHA group than the higher group. In previous studies, an interaction between vitamin D and marine *n*-3 PUFAs was shown. Marine *n*-3 PUFAs and vitamin D3 (cholecalciferol) might synergistically increase 1,25-dihydroxyvitamin D [1,25(OH)2D] levels by inhibiting 24-hydroxylase (a vitamin D-catabolizing enzyme) activities in the kidney and liver, and by upregulating 1a-hydroxylase (a vitamin *D*-activating enzyme) activities in the liver of 5/6 nephrectomy rats [17]. In a clinical study, marine *n*-3 PUFA supplementation significantly increased circulating 1,25(OH)2D levels in dialysis patients [18]. Given that vitamin D insufficiency probably contributes to a poor prognosis [19], this could potentially explain the enhanced effectiveness of vitamin D supplementation in reducing relapse or death in the lower EPA + DHA group since this group had a higher proportion of vitamin D deficiencies.

In this study, the risk of relapse or death was higher in the lower EPA + DHA group than the higher group. This is consistent with the results of a meta-analysis of observational studies showing that individuals with high EPA and DHA levels have a reduced risk of cancer mortality compared to those with lower levels [3]. However, in another study, marine *n*-3 PUFA supplementation did not lead to a reduction in cancer mortality when compared to the placebo [2]. This suggests that a decrease in marine *n*-3 PUFAs is not the cause of cancer mortality but it might be linked to other factors associated with cancer relapse or death. One possible candidate factor is vitamin D. This is because marine *n*-3 PUFAs and vitamin D have similar mechanisms for their anti-tumor effects. In tumor-bearing rats, the administration of *n*-3 PUFA and vitamin D supplementation reduced lipid peroxidation processes in the liver mitochondrial fraction which is an important factor in oncogenesis [20]. Marine *n*-3 PUFA intake has been observed to reduce the risk of occurrence of cancers with abundant regulatory T cell infiltration [21], and vitamin D is also thought to reduce the risk of relapse in the patients with digestive tract cancers with abundant immune cell infiltration [22]. Moreover, although the evidence is not yet conclusive, a couple of meta-analyses of RCTs have suggested a reduction in cancer mortality with vitamin D supplementation [7,8,9,10]. Therefore, marine *n*-3 PUFAs might influence the recurrence or mortality of cancer through an interaction with vitamin D. Indeed, serum EPA + DHA levels had a positive association with 25(OH)D levels in this study. We did not observe a statistically significant correlation between the levels of AA and relapse or mortality. This could be attributed to the fact that blood samples were collected postoperatively, and that metabolism of AA might have occurred during the course of perioperative inflammatory responses, such as wound healing, leading to the absence of a significant correlation.

This study has several limitations. First, this was a post hoc analysis with several missing serum samples (26.7%), resulting in a reduced sample size. However, there were no significant differences in patient characteristics between the two groups. Second, because the analyses assessed a post hoc hypothesis, observer error or bias could have influenced the results. Thus, the findings must be considered exploratory and interpreted with caution. Third, due to multiple analyses, the significance level of *p* < 0.05 might be insufficient and could lead to an increased number of Type I errors. Fourth, since serum PUFA measurements were conducted approximately two weeks postoperatively, the PUFA levels might have changed from their pre-surgery levels. Fifth, since the AMATERASU trial focused on Japanese individuals, the cancer types and diet, particularly fish oil consumption, might differ from other ethnicities. Thus, the study results might not necessarily be generalizable to other populations.

## 5. Conclusions

This post hoc analysis of the AMATERASU trial found that vitamin D supplementation might reduce the risk of relapse or death via an interaction with marine *n*-3 PUFA (EPA + DHA) levels.

## Figures and Tables

**Table 1:** Patient characteristics: vitamin D vs. placebo group.

Characteristic	Patient No. (%) (n = 302)	
	Vitamin D (n = 182)	Placebo (n = 120)
PUFA levels, median (IQR), µg/mL		
Baseline		
Eicosapentaenoic acid (EPA)	42.2 (30.3–62.6)	40.4 (26.1–71.9)
Docosahexaenoic acid (DHA)	107.7 (81–161)	102.5 (84.4–129.9)
EPA + DHA	152.0 (110.5–191.1)	147.7 (111.9–193.0)
Arachidonic acid (AA)	141.3 (119.5–170.5)	142.2 (118.2–166.5)
One year later (n = 185)	(n = 110)	(n = 75)
Eicosapentaenoic acid (EPA)	45.0 (30.0–76.6)	51.1 (34.6–66.2)
Docosahexaenoic acid (DHA)	101.8 (85.7–135.5)	108.2 (81.9–140.9)
EPA + DHA	151.8 (117.1–209.3)	163.2 (119.3–201.6)
Arachidonic acid (AA)	131.1 (111.7–158.6)	134.4 (105.4–169.0)
Sex		
Male	127 (69.8)	77 (64.2)
Female	55 (30.2)	43 (35.8)
Age quartile		
Q1, 35–59	39 (21.4)	31 (25.8)
Q2, 60–65	41 (22.5)	33 (27.5)
Q3, 66–73	51 (28.0)	35 (29.2)
Q4, 74–90	51 (28.0)	21 (17.5)
BMI quartile		
Q1, 15.0–19.7	52 (28.7)	23 (19.3)
Q2, 19.8–21.8	40 (22.1)	33 (27.7)
Q3, 21.9–23.7	44 (24.3)	34 (28.6)
Q4, 23.8–37.3	45 (24.9)	29 (24.4)
Previous history of other cancer	6 (3.3)	7 (5.8)
Comorbidities		
Hypertension	77 (42.3)	46 (38.3)
Diabetes	34 (18.7)	17 (14.2)
Endocrine diseases	23 (12.6)	12 (10.0)
Cardiovascular diseases	15 (8.2)	8 (6.7)
Chronic kidney diseases	4 (2.2)	0 (0)
Asthma	3 (1.7)	0 (0)
Orthopedic diseases	0 (0)	1 (0.8)
Site of cancer		
Esophageal	13 (7.1)	10 (8.3)
Stomach	77 (42.3)	60 (50.0)
Small bowel	0 (0)	1 (0.8)
Colorectal	92 (50.6)	49 (40.8)
Cancer stage		
I	84 (46.2)	51 (42.5)
II	47 (25.8)	37 (30.8)
III	51 (28.0)	32 (26.7)
Pathology		
Adenocarcinoma	166 (91.2)	110 (91.7)
Squamous cell carcinoma	13 (7.1)	9 (7.5)
Other	3 (1.7)	1 (0.8)
Adjuvant chemotherapy	61 (33.5)	45 (37.5)
25(OH)D serum level, ng/mL		
Low: <20	76 (42.0)	52 (43.3)
Middle: ≥20 and ≤40	101 (55.8)	68 (56.7)
High: >40	4 (2.2)	0 (0)
Median (IQR), ng/mL	21 (16–27)	21 (14.5–27)

**Table 2:** Patient characteristics: higher vs. lower EPA + DHA level group.

Characteristic	Patient No. (%) (n = 302)		*p* Value
	Higher Level (n = 151) Median (IQR), µg/mL 192.3 (164.7–231.0)	Lower Level (n = 151) Median (IQR), µg/mL 111.2 (90.3–134.4)	
Intervention Group, No. (%)			0.64
Vitamin D	93 (61.6)	89 (58.9)	
Placebo	58 (38.4)	62 (41.1)	
25(OH)D levels, median (IQR), ng/mL			
Baseline	24 (18–30)	19 (14–24)	<0.0001
One year later in vitamin D group	45.5 (35–57)	40 (32–54)	0.24
One year later in placebo group	23 (18–27)	16 (11.5–23)	0.0006
25(OH)D <20 ng/mL, No. (%)			
Baseline	44 (29.3)	84 (55.6)	<0.001
One year later in vitamin D group	6 (7.3)	5 (6.7)	0.87
One year later in placebo group	18 (34.0)	32 (61.5)	0.005
PUFA levels, median (IQR), µg/mL			
Eicosapentaenoic acid (EPA)	63.5 (48.9–86.2)	29.5 (19.8–37.7)	<0.0001
Docosahexaenoic acid (DHA)	129.8 (115.2–159.1)	82.3 (65.7–94.8)	<0.0001
Arachidonic acid (AA)	155.9 (137.9–179.1)	127.8 (104.7–153.3)	<0.0001
Sex, No. (%)			0.62
Male	100 (66.2)	104 (68.9)	
Female	51 (33.8)	47 (31.1)	
Age quartile, No. (%)			0.04
Q1, 35–59	26 (17.2)	44 (29.1)	
Q2, 60–65	35 (23.2)	39 (25.8)	
Q3, 66–73	51 (33.8)	35 (23.2)	
Q4, 74–90	39 (25.8)	33 (21.9)	
BMI quartile, No. (%)			0.18
Q1, 15.0–19.7	30 (20.1)	45 (29.8)	
Q2, 19.8–21.8	35 (23.5)	38 (25.2)	
Q3, 21.9–23.7	42 (28.2)	36 (23.8)	
Q4, 23.8–37.3	45 (24.9)	32 (21.2)	
Previous history of other cancer, No. (%)	9 (6.0)	4 (2.7)	0.16
Comorbidities, No. (%)			
Hypertension	68 (45.0)	55 (36.4)	0.13
Diabetes	26 (17.2)	25 (16.6)	0.88
Endocrine diseases	19 (12.6)	16 (10.6)	0.59
Cardiovascular diseases	10 (6.6)	13 (8.6)	0.52
Chronic kidney diseases	2 (1.3)	2 (1.3)	1.00
Asthma	0 (0.0)	3 (2.0)	0.08
Orthopedic diseases	0 (0.0)	1 (0.7)	0.32
Site of cancer, No. (%)			0.65
Esophageal	10 (6.6)	13 (8.6)	
Stomach	71 (47.0)	66 (43.7)	
Small bowel	1 (0.7)	0 (0.0)	
Colorectal	69 (45.7)	72 (47.7)	
Cancer stage, No. (%)			0.06
I	77 (51.0)	58 (38.4)	
II	40 (26.5)	44 (29.1)	
III	34 (22.5)	49 (32.5)	
Pathology, No. (%)			
Adenocarcinoma	139 (92.1)	137 (90.7)	0.68
Squamous cell carcinoma	10 (6.6)	12 (8.0)	0.66
Other	2 (1.3)	2 (1.3)	1.00
Adjuvant chemotherapy, No. (%)	47 (31.1)	59 (39.1)	0.15

## Data Availability

The data presented in this study are available on request from the corresponding author Urashima M (urashima@jikei.ac.jp). The data are not publicly available because we want to assess whether the research data from this study aligns with the research interests of other researchers before sharing it.
